# A preliminary preclinical assessment of macromolecular crowding in tissue engineering

**DOI:** 10.1177/00368504251406914

**Published:** 2026-01-21

**Authors:** Kyriakos Spanoudes, Laura Trujillo Cubillo, Stefanie H. Korntner, Diana Gaspar, Dimitrios I. Zeugolis

**Affiliations:** 1 Regenerative, Modular & Developmental Engineering Laboratory (REMODEL) and Science Foundation Ireland (SFI) Centre for Research in Medical Devices (CÚRAM), Biomedical Sciences Building, 8799 University of Galway, Galway, Ireland; 2 Regenerative, Modular & Developmental Engineering Laboratory (REMODEL), Charles Institute of Dermatology, Conway Institute of Biomolecular & Biomedical Research and School of Mechanical & Materials Engineering, University College Dublin (UCD), Dublin, Ireland

**Keywords:** Advanced therapy medicinal products, bone marrow mesenchymal stromal cells, macromolecular crowding, wound healing

## Abstract

**Objectives:**

Although bone marrow mesenchymal stromal cells (BMSCs) are extensively used in biomedicine, they have yet to be used in the commercial development of a tissue engineered medicine. It has been argued that the major roadblock in their commercial deployment is the lengthy *in vitro* culture periods required for the development of implantable tissue surrogates. Macromolecular crowding (MMC) has been shown to enhance and increase extracellular matrix deposition in eukaryotic cell culture, allowing for the accelerated development of tissue facsimiles.

**Methods:**

With these in mind, human BMSCs were cultured under MMC conditions and the developed tissue-engineered medicine was assessed *in vitro* and *in vivo* in a humanised athymic nude mouse excisional wound splinting model.

**Results:**

Starting with basic cell function analysis, MMC did not significantly affect cell metabolic activity, viability and proliferation. Electrophoresis and immunofluorescence analyses revealed that MMC significantly increased collagen type I and collagen type IV deposition, without significantly affecting collagen type III deposition. Flow cytometry analysis demonstrated similar CD44, CD73, CD90, CD146, HLA-ABC, CD31, CD45, CD80 and CD86 expression between the without and the with MMC groups. Interestingly though the MMC group had higher CD105 and lower HLA-DR expression than the without MMC group. Preclinical analysis revealed similar wound closure, scar index and epidermal thickness between the without and the with MMC groups, largely attributed to issues encountered with the model.

**Conclusions:**

Overall, this preliminary study demonstrates the potential of MMC in the accelerated development of functional and extracellular matrix-rich human BMSC-based tissue-engineered medicines.

## Introduction

Bone marrow mesenchymal stromal cells (BMSCs) are by far the most studied adult stromal cell population in medicine (Source: PubMed; Term searched: bone marrow cells, 318,130 results, as opposed to 82,467 results for Term searched: adipose cells; Date: 27/09/2024), with numerous ongoing clinical trials (Source: clinicaltrials.gov; Term searched in Intervention/treatment: bone marrow cells, 2195 results, as opposed to 861 results for Term searched in Intervention/treatment: adipose cells; Date: 27/09/2024) for a diverse range of clinical indications. One should also note that their safety, efficacy and efficiency have been well-documented in the literature, in both preclinical and clinical settings for a diverse range of dermatological conditions. For example, in preclinical setting, intradermal injections have shown promise in full-thickness incisional acute cutaneous wounds in pigs^1^ and in full-thickness burn wounds in rats.^2^ Using a collagen sponge as a carrier, transplanted cells resulted in regeneration of subcutaneous tissue in nude mice and skin healing in 18 patients with intractable dermatopathies.^3^ In a randomised, blinded placebo-controlled trial, intradermal transplantation of autologous BMSCs was proven to be a safe and effective strategy to promote mechanical stretch induced skin regeneration.^4^ In a case report, where a decellularised allogeneic dermal matrix / autologous BMSCs / autologous split-skin graft was compared to a decellularised allogeneic dermal matrix / autologous split-skin graft, the BMSC transplant resulted in significantly less contraction in a patient who was severely burned.^5^ In another case study, allogenic BMSCs were shown to accelerate recovery of homeostasis and to promote healing of a patient with deep skin burns.^6^ Despite all these positive patient therapeutic outcomes, no BMSC-based therapy is currently available in the wound healing space.

Extracellular matrix (ECM) is a depot of bioactive molecules that through a spatiotemporal dynamic crosstalk with surrounding cells promotes tissue remodelling and wound healing post injury.^7–12^ Commercially available living skin substitutes require prolonged *ex vivo* culture for ECM deposition (e.g. Apligraf^®^ 17–20 days,^13^ denovoSkin™ 21–31 days^14^). To avoid phenotypic drift, BMSC-based medicines are transplanted immediately after cell expansion without any cell deposited ECM. In the absence of cell deposited ECM, the secretome, and therefore, the therapeutic potential, of the BMSCs is not fully exploited, rendering the potential therapy inferior and more expensive to the state-of-the-art. To substantiate this one should consider that adipose derived MSC secretome has been shown to lead to diabetic wound healing in rats by modulating angiogenesis, scarring and inflammation.^15^ In a full-thickness rat skin excision model although both adipose-derived MSCs and adipose derived MSCs’ secretome enhanced early phase wound healing, the secretome group promoted fibroblast proliferation and migration and suppressed inflammation.^16^ In a diabetic rat splinted wound healing model, human foetal MSC secretome, delivered through poly lactic-co-glycolic acid particles, significantly enhanced wound healing via improved vascularisation and suppressed inflammation.^17^ In clinical setting, application of cultured (scraped cell layers as opposed to injected cell suspensions) autologous BMSCs resulted in complete healing and dermal rebuilding in three patients with chronic wounds that had not closed even after multiple applications of Apligraf^®^ or autologous skin grafting.^18^ It is evidenced that for superior patient therapeutic outcomes, the MSC secretome must be part of the intervention strategy. Upon this supposition, the concept of utilising macromolecular crowding (MMC) in tissue engineering was conceived.

In eukaryotic cell culture, MMC is defined as the addition of macromolecules in the culture media that by decreasing diffusion via restriction of molecular motion, favour protein–substrate interactions and result in enhanced and accelerated ECM deposition.^19–21^ To-date, various MMC agents (e.g. carrageenan,^22–25^ dextran sulphate,^26^ polyvinylpyrrolidone,^27^ polysucrose cocktails,^28–32^ polysucrose / dextran sulphate cocktail^33^) have been assessed in human BMSC cultures. Although MMC has shown promise in preclinical setting (one study with human adipose derived MSCs cultured with carrageenan,^34^ one study with decellularised matrices derived from human BMSCs cultured with carrageenan^35^ and one more study with decellularised matrices derived from human umbilical cord blood MSCs cultured with polysucrose^36^), not one study has assessed *in vivo* a tissue-engineered medicine produced with human BMSCs under MMC conditions.

Herein, we ventured to assess the potential of a tissue-engineered medicine, composed of human BMSCs that were cultured under MMC conditions in wound healing context. We selected carrageenan as MMC agent, as, due to its negative charge and high polydispersity, it induces the highest ECM deposition in the shortest period of time.^37,38^ A humanised athymic nude mouse excisional wound splinting model was used, as its suitability for human MSC transplantation is well-documented in the literature.^39–42^

## Materials and methods

Tissue culture consumables were purchased from Sarstedt (Ireland) and NUNC (Denmark). All chemicals, cell culture media and reagents were purchased from Sigma-Aldrich (Ireland), unless otherwise stated.

### Cell isolation and culture

Fresh human bone marrow from the iliac crest was purchased from Lonza (UK) and human BMSCs were isolated and cultured in basal medium [α-minimal essential medium, GlutaMax™, ThermoFisher Scientific, Ireland) supplemented with 10% foetal bovine serum (FBS) and 1% penicillin / streptomycin], following established protocols.^22,24,30^ A total of 50,000 human BMSCs per cm^2^ were seeded at passage 4 in basal medium and after 24 h, the media were changed to media with 100 µM of l-ascorbic acid 2-phosphate sesquimagnesium salt hydrate without (-) / with (+) MMC (carrageenan at 75 µg/mL). The cells were cultured for 5 days for *in vitro* analysis and prior to *in vivo* transplantation as cell sheets.

### Cell metabolic activity, viability and proliferation analyses

Using established protocols,^43–45^ after 5 days of culture BMSCs-MMC and BMSCs + MMC were assessed for metabolic activity using the alamarBlue™ (Invitrogen, UK) assay; for viability using the Live/Dead™ assay with calcein AM (ThermoFisher Scientific, Ireland) and ethidium homodimer I (ThermoFisher Scientific, Ireland) stainings; and for proliferation using 4′,6-diamidino-2-phenylindole (DAPI, Invitrogen, Ireland) stained cell layers. Images were captured with an Olympus IX-81 (Olympus Corporation, Japan) inverted fluorescence microscope and processed with ImageJ (NIH, USA).

### Collagen deposition analysis via electrophoresis and immunofluorescence

Collagen type I deposition at day 5 was assessed using non-reduced sodium dodecyl sulphate – polyacrylamide gel electrophoresis (SDS-PAGE), as per established protocol.^46^ In brief, 100 μg/mL collagen type I standard (Symatese Biomateriaux, France) in pepsin in acetic acid (standard) and cell layers in pepsin in acetic acid were neutralised, denatured, loaded and separated on a Mini-Protean^®^ 3 system (Bio-Rad Laboratories, UK). Protein bands were stained with SilverQuest™ kit (ThermoFisher Scientific, UK) according to the manufacturer's protocol. Using ImageJ (NIH, USA), the densities of collagen α1(I) and collagen α2(I) chains of the cell layers were quantified and correlated to the densities of collagen α1(I) and collagen α2(I) chains of the standard.

At day 5, collagen type I, collagen type III and collagen type IV deposition was quantified using established immunofluorescence protocols.^47,48^ In brief, cell layers were washed, fixed, blocked, incubated with mouse anti-collagen type I (NB600-408, Novus Biologicals, USA), rabbit anti-collagen type III (Ab7778, Abcam, Ireland) or rabbit anti-collagen type V (Ab7046, Abcam, Ireland) primary antibodies, washed, incubated with AlexaFluor^®^ 488 goat anti-rabbit (A11008, Invitrogen, Ireland) or AlexaFluor^®^ 488 goat anti-mouse (A10667, Invitrogen, Ireland) secondary antibodies, washed and stained with DAPI (Invitrogen, Ireland). Images were captured with an Olympus IX-81 (Olympus Corporation, Japan) inverted fluorescence microscope and processed with ImageJ (NIH, USA).

### Flow cytometry analysis

Flow cytometry was conducted at day 5 using standard protocols^22,30^ for positive [CD44 (BD Bioscience, Ireland), CD73 (BD Bioscience, Ireland), CD90 (BD Bioscience, Ireland), CD105 (BD Bioscience, Ireland), CD146 (Invitrogen, Ireland), HLA-ABC (ThermoFisher Scientific, Ireland)] and negative [CD31 (Invitrogen, Ireland), CD45 (Invitrogen, Ireland), CD80 (Invitrogen, Ireland), CD86 (BD Bioscience, Ireland) and HLA-DR (ThermoFisher Scientific, Ireland)] markers. Briefly, cells were detached, centrifuged and resuspended in 2% FBS in phosphate-buffered saline (PBS). Following straining, the cells were counted, diluted, stained with the fluorochrome-labelled antibodies for 30 min at 4°C, washed with PBS and resuspended in 2% FBS in PBS. Unstained cell samples were used to correct for background autofluorescence and SYTOX™ Blue Dead Cell Stain (Invitrogen, UK) was used to label and exclude dead cells. Single stained samples; fluorescence minus one controls; and isotype control antibodies were used to determine the level of spectral overlap between different fluorophores and for compensation; to determine gating boundaries; and to assess the level of background staining and non-specific binding, respectively. The analysis was conducted using a BD FACSCanto™ II cytometer (BD Biosciences, UK). The median fluorescence intensity was calculated using the FlowJo^®^ software v10 (TreeStar Inc., USA). For gating, a primary gate was placed on the area versus height signal of the forward scatter dot plot to discriminate for doublets and cell aggregates. The single cell population was identified by defining the gated population on a side scatter area signal versus a forward scatter area signal dot plot. Single parameter histograms were generated, overlayed with respective isotype controls and range gates were used to determine the % of cells expressing the individual surface markers.

### Preclinical analysis

All animal experiments and procedures were approved by the Animal Care and Research Ethics Committee of the University of Galway and the Irish Health Products Regulatory Authority (Licence Number: AE 19125/P051 K) and were conducted as per Irish laws governing laboratory animal experimentation. The splinted wound healing model and subsequent analyses were selected based on previous publications.^34,39,49,50^ In brief, female athymic nude mice (7 weeks old) were purchased from Charles River (Ireland). After one week acclimatisation, the animals were randomly assigned to the following groups: non-treated control, human BMSCs without MMC (BMSCs-MMC) and human BMSCs with MMC (BMSCs + MMC). The human BMSC groups were delivered (per wound) as a single dose and single cell sheet that was produced after 5 days of culture of 50,000 human BMSCs per cm^2^. Two circular (5 mm in diameter) full-thickness (epidermis, dermis, subcutaneous tissue and panniculus carnosus muscle) wounds were created with a single puncture and a silicone splint was sutured around each wound to prevent contraction and promote healing by epithelisation. Each animal received the same treatment in both wounds and all animals received identical post-operative pharmacological treatment. Wound closure rate was determined by taking digital pictures of the wounds with a digital camera (Canon, Japan) at the different time points and processing them with ImageJ (NIH, USA). The % wound closure was calculated as follows: [(area of original wound – area of actual wound) / area of original wound]×100. Harvested skin tissue samples were fixed with 4% paraformaldehyde and paraffin embedded. After sectioning (5 μm in thickness), deparaffinisation (2 immersions in xylene) and re-hydrations in descending concentrations of ethanol (100%, 90%, 70% and 0% in distilled water), the sections were stained using haematoxylin-eosin stain and Masson–Goldner's trichrome stain (both Carl Roth, Germany). Images were captured using an Olympus VS120 virtual slide microscope and processed using the OlyVIA software (both Olympus Corporation, Japan). Deparaffinised sections were blocked, incubated with the primary rabbit anti-cytokeratin 5 (Abcam, UK) antibody, washed, incubated with the secondary biotinylated swine anti-rabbit (Dako, USA) antibody and treated with ABC horseradish peroxidase labelled Vectastain Elite ABC reagent (Vector, UK), diaminobenzidine (Dako, UK) and haematoxylin. Images were captured using an Olympus VS120 virtual slide microscope and processed using the OlyVIA software (both Olympus Corporation, Japan). Scar index [scar index (μm) = scar area (μm^2^) / average dermal thickness (μm)] and epidermal thickness were evaluated using ImageJ software (NIH, USA) on images from Masson–Goldner's trichrome stained histological sections.

### Statistical analysis

Data are expressed as mean ± standard deviation. Number of replicates is indicated in each figure legend. Statistical analysis was performed using either MINITAB^®^ (Minitab LLC., USA) or Prism (GraphPad Software, USA). One-way analysis of variance was used for multiple comparisons and Tukey's post hoc test was used for pairwise comparisons after confirming the samples followed a normal distribution (Anderson–Darling test) and had equal variances (Bartlett's and Levene's test for homogeneity of variances). When either or both assumptions were violated, non-parametric analysis was conducted using Kruskal–Wallis test for multiple comparisons and Mann–Whitney test for pairwise comparisons. Statistical significance was accepted at *p* < 0.05.

## Results

### Cell metabolic activity, viability and proliferation analyses

**Figure 1.**
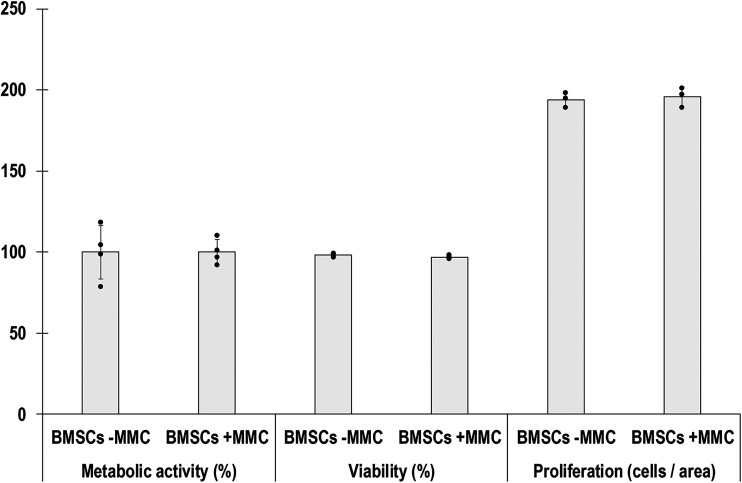
Quantitative analysis of % metabolic activity, viability and proliferation of human BMSCs after 5 days in culture without (−) and with (+) MMC. *N* = 4 for metabolic activity and *N* = 3 for viability and proliferation.

Cell metabolic activity (Figure 1A), viability (Figure 1B) and proliferation (Figure 1C) analyses showed no significant (*p* > 0.05) differences between the groups.

### Collagen deposition analysis via electrophoresis and immunofluorescence

**Figure 2.**
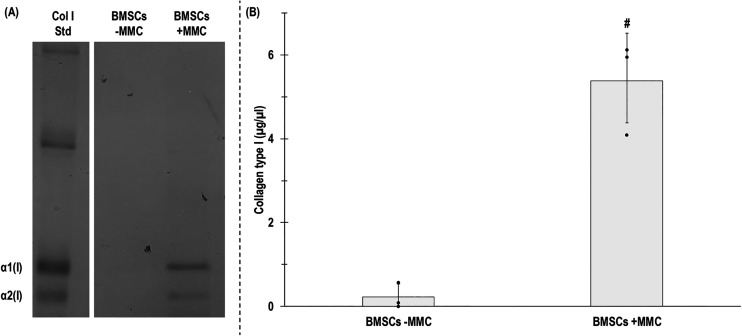
SDS-PAGE (A) and densitometry analysis (B) of collagen type I deposition of human BMSCs after 5 days in culture without (−) and with (+) MMC. # indicates significant (*p* < 0.05) difference. *N* = 3.

**Figure 3.**
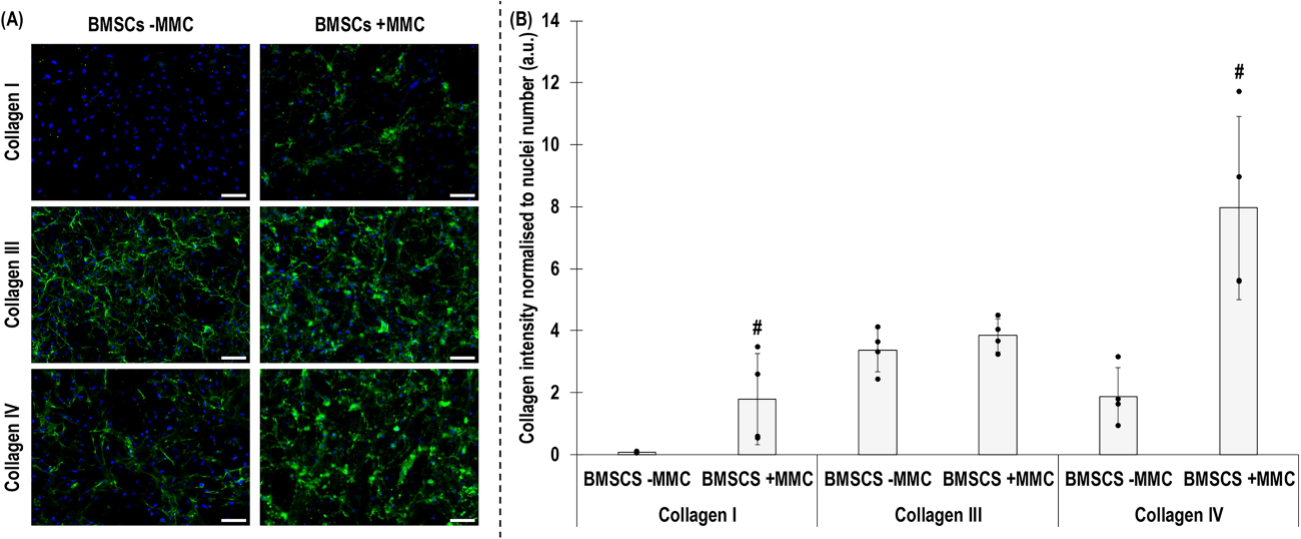
Immunofluorescence (A) and normalised to cell number image intensity analysis (B) of collagen type I, collagen type III and collagen type IV deposition of human BMSCs after 5 days in culture without (−) and with (+) MMC. # indicates significant (*p* < 0.05) difference. Scale bar: 100 μm. *N* = 4.

The SDS-PAGE and densitometry analyses showed that +MMC significantly (*p* < 0.05) increased collagen type I deposition (Figure 2). Immunofluorescence and fluorescence intensity analyses revealed that +MMC significantly (*p* < 0.05) increased collagen type I and collagen type IV deposition, without affecting (*p* > 0.05) collagen type III deposition (Figure 3).

### Flow cytometry analysis

**Figure 4.**
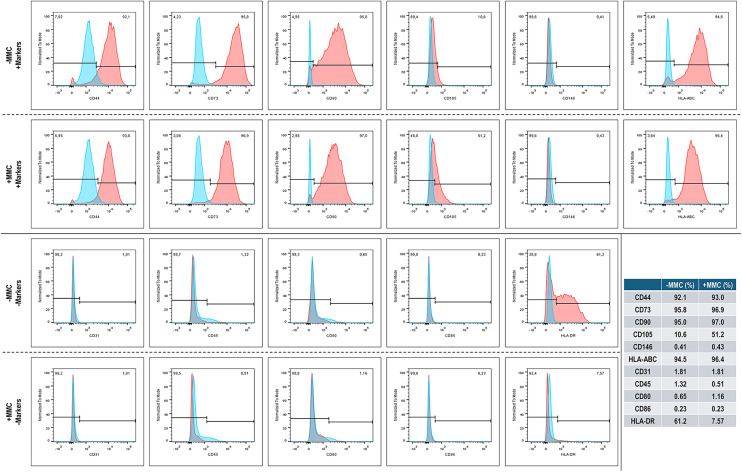
Flow cytometry analysis of human BMSCs after 5 days in culture without (−) and with (+) MMC. Blue: isotype control. Red: antibody. *N* = 1.

Flow cytometry analysis (Figure 4) revealed that over 92% of the BMSCs-MMC and the BMSCs + MMC were positive for the positive MSC markers CD44 (92.1% and 93.0%, respectively), CD73 (95.8% and 96.9%, respectively), CD90 (95.0% and 97.0%, respectively) and HLA-ABC (94.5% and 96.4%, respectively). The BMSCs-MMC and the BMSCs + MMC showed variable results for the positive MSC marker CD105 (10.6% and 51.2%, respectively, were positive) and less than 1% expressed the positive MSC marker CD146 (0.41% and 0.43%, respectively, were positive).

Further, less than 2% of the BMSCs-MMC and the BMSCs + MMC were positive for the negative MSC markers CD31 (1.81% and 1.81%, respectively), CD45 (1.32% and 0.51%, respectively), CD80 (0.65% and 1.16%, respectively) and CD86 (0.23% and 0.23%, respectively). The BMSCs-MMC and the BMSCs + MMC showed variable results for the negative MSC marker HLA-DR (61.2% and 7.57%, respectively, were positive).

### Wound closure, histological and immunohistochemical analyses

**Figure 5.**
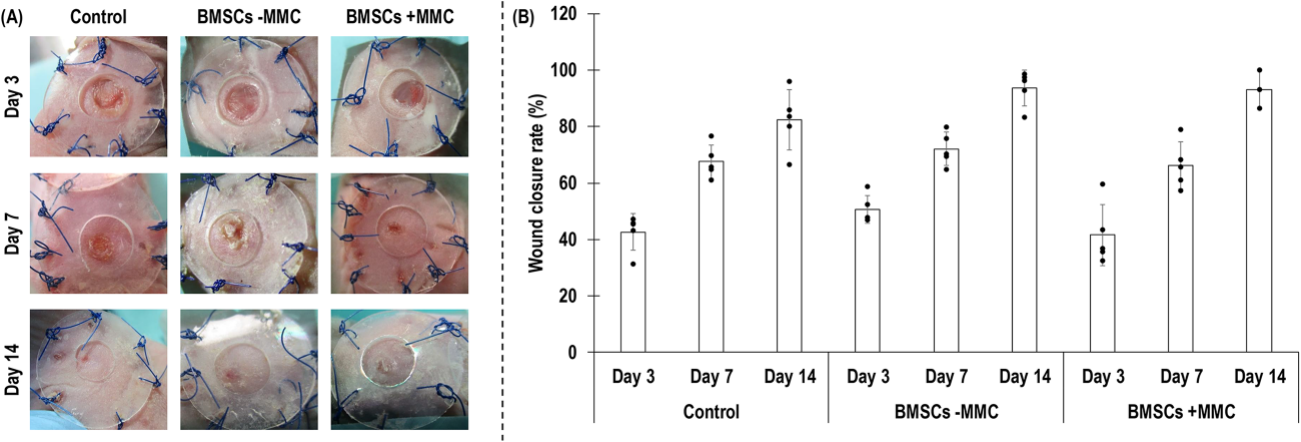
Qualitative (A) and quantitative (B) wound closure analyses at day 3, day 7 and day 14 of non-treated control group and treated with human BMSCs after 5 days in culture without (−) and with (+) MMC test groups. *N* = 5 wounds for all but BMSCs at day 14, for which *N* = 3 wounds.

**Figure 6.**
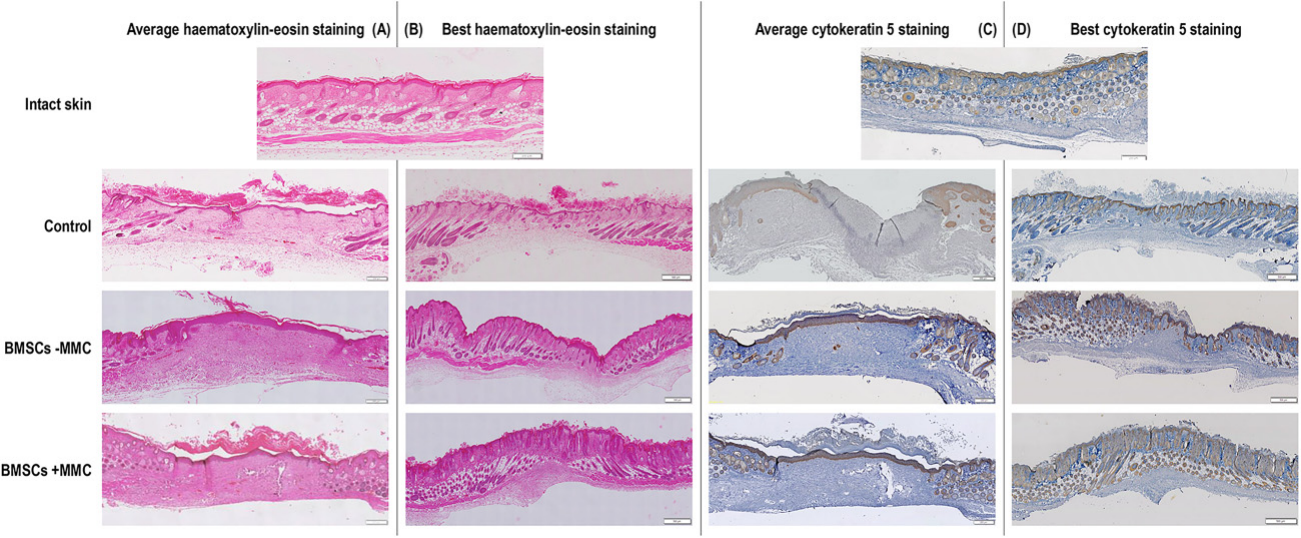
Qualitative haematoxylin–eosin (A, B) and cytokeratin 5 (C, D) stainings of intact skin and of day 14 post-surgery of non-treated control group and treated with human BMSCs after 5 days in culture without (−) and with (+) MMC test groups. Scale bars: 200 μm for intact skin, average haematoxylin–eosin and average cytokeratin 5 images; 500 μm for best haematoxylin–eosin and best cytokeratin 5 images. *N* = 5 wounds for all groups for haematoxylin–eosin. *N* = 1, 4, 6 and 5 wounds for intact skin, control, BMSCs-MMC and BMSCs + MMC, respectively, for cytokeratin 5.

**Figure 7.**
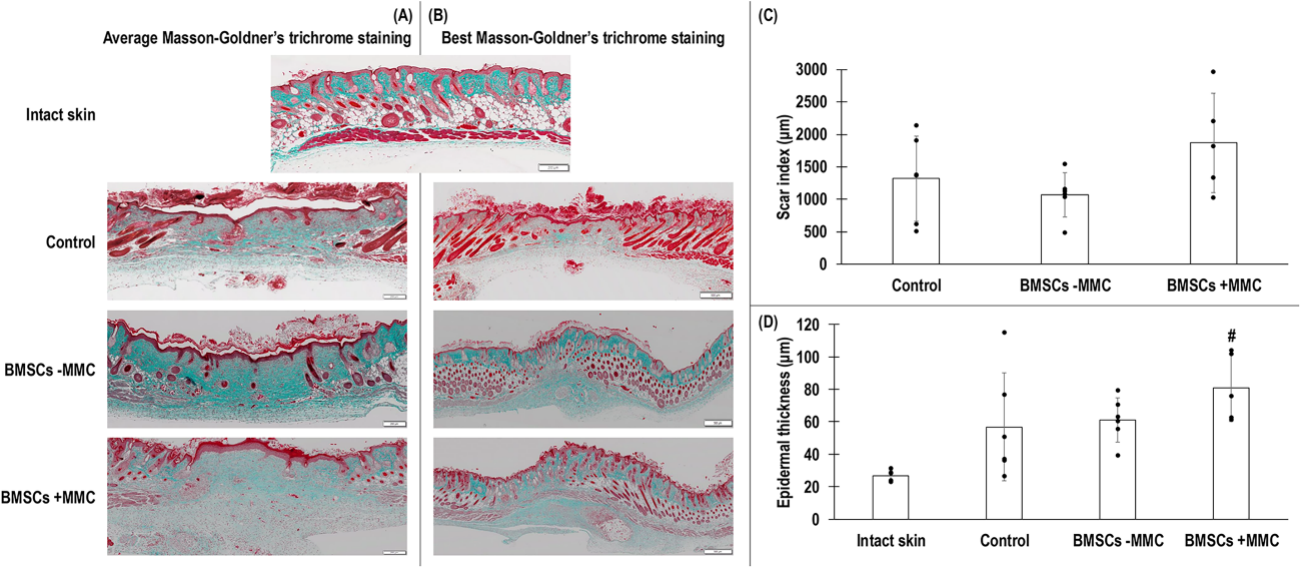
Qualitative Masson–Goldner’s trichrome staining (A, B) and quantitative scar index (C) and epidermal thickness (D) analyses of intact skin and of day 14 post-surgery of non-treated control group and treated with human BMSCs after 5 days in culture without (−) and with (+) MMC test groups. # indicates significant (*p* < 0.05) difference. Scale bars: 200 μm for intact skin and average Masson–Goldner's trichrome images; 500 μm for best Masson–Goldner's trichrome images. *N* = 1, 6, 6 and 5 wounds for intact skin, control, BMSCs-MMC and BMSCs + MMC, respectively, for Masson–Goldner's trichrome. *N* = 6, 6 and 5 wounds for control, BMSCs-MMC and BMSCs + MMC, respectively, for scar index. *N* = 5, 6, 6 and 5 wounds for intact skin, control, BMSCs-MMC and BMSCs + MMC, respectively, for epidermal thickness.

Neither infection nor necrotic tissues were found in any of the groups at any time point. The animals were removing the splints, which had to be re-sutured several times during the course of the study. Qualitative (Figure 5A) and quantitative (Figure 5B) wound closure analysis revealed no significant (*p* > 0.05) difference between the groups at a given time point. Haematoxylin–eosin staining showed complete re-epithelisation in all groups 14 days after injury and immunohistochemical analysis of cytokeratin 5 revealed that in all groups, protein expression was restricted to the epidermal layers and hair follicles (Figure 6). Further, at best case scenario, haematoxylin–eosin and cytokeratin 5 stainings revealed that the BMSC treated groups outperformed the empty defect control group (Figure 6). Masson–Goldner's trichrome staining revealed dense collagenous tissue formation in the BMSCs ± MMC, but not in the control group; scar index analysis revealed no significant (*p* > 0.05) differences between the groups; and epidermal thickness analysis revealed only the BMSCs + MMC group to have significantly (*p* < 0.05) higher epidermal thickness compared to the intact skin group (Figure 7). Further, at best case scenario, Masson–Goldner's trichrome staining revealed that the BMSC treated groups outperformed the empty defect control group (Figure 7).

## Discussion

Although human BMSCs have shown promise for a range of clinical indications, including wound healing, no BMSC-based tissue-engineered medicine has been commercial reality to-date. This limited technology transfer is attributed to the prolonged *in vitro* cultured periods required to develop an ECM / secretome-rich implantable construct that are associated with phenotypic drift and high manufacturing costs and therefore high cost of goods for healthcare providers. Although MMC has been shown to enhance and accelerate ECM deposition in various cell types, including BMSCs, a tissue-engineered construct based on BMSCs and MMC has yet to be assessed in preclinical setting. Herein, we assessed the therapeutic potential of human BMSCs, which were cultured -MMC and + MMC conditions, in a humanised athymic nude mouse excisional wound splinting model.

### Cell metabolic activity, viability and proliferation analyses

Starting with basic cell function analysis, no differences were observed between the groups in metabolic activity, viability and proliferation. To-date carrageenan has been used extensively as MMC agent in a diverse range of cell populations (e.g. skin fibroblasts,^38,51,52^ human tenocytes,^45,48,53^ human BMSCs,^23^ human adipose derived MSCs^23,47,54^ and human umbilical cord MSCs^43^) with no study reporting any negative effect. One should also note that carrageenan-based devices have long positive history in clinical setting,^55–57^ further verifying its safety, efficiency and efficacy.

### Collagen deposition analysis via electrophoresis and immunofluorescence

Extracellular matrix molecules are essential players in both physiological and pathological wound healing.^58^ Therefore, the development of a tissue-engineered medicine, which accounts for physiological levels of ECM molecules, is crucial for functional tissue repair and regeneration. Collagen type I, collagen type III and collagen type IV are essential ECM molecules in wound healing. Collagen type III has various key roles (provides a temporary wound matrix, increases tensile strength, facilitates collagen type I fibrillogenesis) in the early stages of wound healing.^59^ Collagen type I replaces collagen type III as the wound healing progresses and confers to the regenerated tissue mechanical stability.^60^ Collagen type IV is essential for basement membrane formation.^61^ Under MMC conditions, a substantial increase in collagen type I and collagen type IV deposition was observed, whilst the amount of deposited collagen type III was not affected, as per previously published work.^23^ It is interesting to note that other MMC agents (e.g. Ficoll^®^,^45^^62–64^ hyaluronic acid^47,65^) have resulted in increased collagen type III deposition. Although the mechanism that induces this selective collagen types increased deposition has yet to be elucidated, we consider the indifference in collagen type III deposition of therapeutic value, as increased collagen type III is often associated with fibrosis.^66–68^

### Flow cytometry analysis

Flow cytometry analysis made apparent that over 90% of the -MMC and + MMC BMSC populations expressed the positive MSC markers CD44, CD73, CD90 and HLA-ABC and less than 2% of the -MMC and + MMC BMSC populations expressed the negative MSC markers CD31, CD45, CD80 and CD86, as one would have expected.^69–72^ Nonetheless, BMSCs-MMC (10.6% were positive) and, to a lesser extent, BMSCs + MMC (51.2% were positive) had low expression of the positive marker CD105. Although CD105 is widely accepted as MSC marker,^69,73^ it is worth noting that CD105 negative populations have shown typical MSC differentiation potential and immunomodulatory capacity^74^ and CD105 expression has been shown to depend on passaging and culture media composition.^75–78^ With respect to the positive MSC marker CD146, both groups were almost entirely negative (less than 0.5% positive). Again, although CD146 is commonly considered as a positive MSCs marker^79,80^ and CD146 positive cells have been shown to display greater therapeutic potential than CD146 negative cells in arthritis,^81^ several studies have shown similar differentiation potential for both CD146 negative and positive MSC populations,^82–85^ with some differences though in modulating immunological and proliferation properties (improved for the CD146 positive cells).^86^ With regards to the negative MSC marker HLA-DR, a fairly high % of the BMSCs-MMC were positive (61.2%) and a relatively low % of the BMSCs + MMC were positive (7.57%). Again, although MSCs should be negative for HLA-DR,^69^ *in vitro* expression of HLA-DR has been reported to be donor- and culture media-dependent.^87–90^ Some studies have even considered it an obsolete marker,^87^ considering its expression informative, but not critical.^91^ Collectively, we believe that the implanted cells had maintained their MSC function and differences in the expression of surface markers may be attributed to donor and media variations between studies, as explained earlier. The differences between the -MMC and + MMC groups (for CD105, higher % of positive cells in the + MMC group, and HLA-DR, lower % of positive cells in the + MMC group) could be attributed to the enhanced ECM deposition that better protected the cells. To substantiate this one should consider that cells on stiff two-dimensional substrates (i.e. cells grown on tissue culture plastic under -MMC conditions) are more spread than cells on soft three-dimensional substrates (i.e. cells grown on deposited ECM under + MMC conditions)^92,93^ and the rebound level of CD105 has been shown to be high when the cell spreading area is small (i.e. under + MMC conditions).^94^ With respect to HLA-DR, it is known that its expression is associated with implant / cell rejection and immune response,^95–98^ thus its reduction under + MMC conditions is welcome. In any case though, one should note that even clinical-grade BMSCs have been found positive for HLA-DR.^91^ Although the specific mechanism is unclear at this stage, considering that various growth factors have been shown to affect HLA-DR expression,^99–101^ we speculate that this reduction under + MMC conditions may be due to growth factor retention in the deposited ECM.

### Wound closure, histological and immunohistochemical analyses

The use of MSCs for skin tissue engineering has been advocated due to their immunomodulatory, anti-inflammatory, mitogenic and angiogenic capacities, along with their rich secretome.^102–105^ Various studies have demonstrated human BMSCs-based^106,107^ and MMC-based^34–36^ therapies to result in improved healing; however, this was not witnessed herein. Indeed, preclinical analysis revealed similar wound closure, re-epithelisation, scar index and epidermal thickness between the groups. It should be noted that negative outcomes (e.g. infection, inflammation, necrosis) were not observed. Further, herein we transplanted a single cell sheet per wound that was produced after 5 days in culture (without or with MMC) using 50,000 human BMSCs per cm^2^ as original seeding density; other studies that have demonstrated efficiency and efficacy of stem cell sheets used substantially higher cell numbers (e.g. 2,000,000 human BMSCs per cm^2108^; 3 layers of 300,000 human adipose derived mesenchymal stromal cells per cm^2^ per layer^109^; ∼350,000 human amniotic fluid mesenchymal stromal cells per cm^2110^). This indifference between the groups may also be due to the animals removing the silicone rings, which resulted in healing by tissue contraction. Similar indifference between the groups has been reported previously.^111,112^ The reader should note that over the years various modifications of the model (e.g. the use of Elizabethan collars and jackets with plastic wings^113^ or wound chambers^114^ or shape memory alloys as internal splints^115^) have been suggested. Regretfully, these approaches were not in our licence and therefore we were not able to implement them. Researchers should point out such crucial limitations of preclinical experimentation to inform future preclinical endeavours. Alternative models, such as a full-thickness Sprague–Dawley rat model of 1.0 cm by 1.0 cm wound area^116^ or a full-thickness athymic nude mouse, nu/nu, model of 2.5 cm by 2.5 cm wound area,^117^ should also be considered.

## Conclusions

Despite the fact that human BMSCs are extensively used in tissue engineering and regenerative medicine, they have yet to constitute the building blocks of a commercially available tissue-engineered medicine. This limited technology transfer from research to development has been attributed to the protracted culture periods required to develop a tissue-engineered medicine that are associated with phenotypic drift, loss of therapeutic potential and very high manufacturing costs. Considering that MMC has the potential to accelerate the development of tissue-engineered medicines, herein human BMSCs were cultured under MMC conditions and the developed tissue-engineered medicine was assessed *in vitro* and *in vivo*. Our *in vitro* data indicate that MMC does not affect human BMSC function and enhances and accelerates ECM deposition. Regretfully, the *in vivo* data are inconclusive due to issues encountered with the model. In any case, this preliminary study sets the foundations for exploiting MMC in the development of functional and extracellular matrix-rich tissue-engineered medicines.

## References

[1] Ochiai H Kishi K Kubota Y , et al. Transplanted mesenchymal stem cells are effective for skin regeneration in acute cutaneous wounds of pigs. Regen Ther 2017; 7: 8–16. 30271847 10.1016/j.reth.2017.06.003 PMC6134893

[2] El-Sayed M Atwa A Sofy A , et al. Mesenchymal stem cell transplantation in burn wound healing: uncovering the mechanisms of local regeneration and tissue repair. Histochem Cell Biol 2024; 161: 165–181. 37847258 10.1007/s00418-023-02244-y PMC10822811

[3] Yoshikawa T Mitsuno H Nonaka I , et al. Wound therapy by marrow mesenchymal cell transplantation. Plast Reconstr Surg 2008; 121: 860–877. 18317135 10.1097/01.prs.0000299922.96006.24

[4] Zhou S Zhang G Xie Y , et al. Autologous stem cell transplantation promotes mechanical stretch induced skin regeneration: a randomized phase I/II clinical trial. EBioMedicine 2016; 13: 356–364. 27876353 10.1016/j.ebiom.2016.09.031 PMC5264315

[5] Xu Y Huang S Fu X . Autologous transplantation of bone marrow-derived mesenchymal stem cells: a promising therapeutic strategy for prevention of skin-graft contraction. Clin Exp Dermatol 2012; 37: 497–500. 22300217 10.1111/j.1365-2230.2011.04260.x

[6] Rasulov M Vasilchenkov A Onishchenko N , et al. First experience of the use bone marrow mesenchymal stem cells for the treatment of a patient with deep skin burns. Bull Exp Biol Med 2005; 139: 141–144. 16142297 10.1007/s10517-005-0232-3

[7] Zimmerlin L Park T Zambidis E , et al. Mesenchymal stem cell secretome and regenerative therapy after cancer. Biochimie 2013; 95: 2235–2245. 23747841 10.1016/j.biochi.2013.05.010 PMC3825748

[8] González-González A García-Sánchez D Dotta M , et al. Mesenchymal stem cells secretome: the cornerstone of cell-free regenerative medicine. World J Stem Cells 2020; 12: 1529–1552. 33505599 10.4252/wjsc.v12.i12.1529 PMC7789121

[9] Daneshmandi L Shah S Jafari T , et al. Emergence of the stem cell secretome in regenerative engineering. Trends Biotechnol 2020; 38: 1373–1384. 32622558 10.1016/j.tibtech.2020.04.013 PMC7666064

[10] Gwam C Mohammed N Ma X . Stem cell secretome, regeneration, and clinical translation: a narrative review. Ann Transl Med 2021; 9: 70. 33553363 10.21037/atm-20-5030 PMC7859812

[11] Raghow R . The role of extracellular matrix in postinflammatory wound healing and fibrosis. FASEB J 1994; 8: 823–831. 8070631 10.1096/fasebj.8.11.8070631

[12] Potekaev N Borzykh O Medvedev G , et al. The role of extracellular matrix in skin wound healing. J Clin Med 2021; 10: 5947. 34945243 10.3390/jcm10245947 PMC8706213

[13] Eaglstein W Falanga V . Tissue engineering and the development of Apligraf, a human skin equivalent. Clin Ther 1997; 19: 894–905. 9385478 10.1016/s0149-2918(97)80043-4

[14] CUTISS. Personalized, bio-engineered skin grafts for the permanent treatment of skin defects (denovoSkin™). 2021. https://cutiss.swiss

[15] Izadi R Hejazi S Bahramikia S . Alternative viewpoint against diabetic wound based on stem cell secretome that can mediated angiogenesis and reduce inflammation. Arch Dermatol Res 2023; 316: 28. 38060015 10.1007/s00403-023-02739-7

[16] Ma H Lam P Siu W , et al. Adipose tissue-derived mesenchymal stem cells (ADMSCs) and ADMSC-derived secretome expedited wound healing in a rodent model - A preliminary study. Clin Cosmet Investig Dermatol 2021; 14: 753–764. 10.2147/CCID.S298105 PMC8255652 34234501

[17] Wang B Pang M Song Y , et al. Human fetal mesenchymal stem cells secretome promotes scarless diabetic wound healing through heat-shock protein family. Bioeng Transl Med 2022; 8: e10354. 10.1002/btm2.10354 PMC9842061 36684113

[18] Badiavas E Falanga V . Treatment of chronic wounds with bone marrow-derived cells. Arch Dermatol 2003; 139: 510–516. 12707099 10.1001/archderm.139.4.510

[19] Raghunath M Zeugolis D . Transforming eukaryotic cell culture with macromolecular crowding. Trends Biochem Sci 2021; 46: 805–811. 33994289 10.1016/j.tibs.2021.04.006

[20] Tsiapalis D Zeugolis D . It is time to crowd your cell culture media – Physicochemical considerations with biological consequences. Biomaterials 2021; 275: 120943. 34139505 10.1016/j.biomaterials.2021.120943

[21] Zeugolis D . Bioinspired in vitro microenvironments to control cell fate: focus on macromolecular crowding. Am J Physiol Cell Physiol 2021; 320: C842–C849. 10.1152/ajpcell.00380.2020 33656930

[22] Cigognini D Gaspar D Kumar P , et al. Macromolecular crowding meets oxygen tension in human mesenchymal stem cell culture – A step closer to physiologically relevant in vitro organogenesis. Sci Rep 2016; 6: 30746. 27478033 10.1038/srep30746 PMC4967872

[23] Gaspar D Ryan C Zeugolis D . Multifactorial bottom-up bioengineering approaches for the development of living tissue substitutes. FASEB J 2019; 33: 5741–5754. 30681885 10.1096/fj.201802451R

[24] Graceffa V Zeugolis D . Carrageenan enhances chondrogenesis and osteogenesis in human bone marrow stem cell culture. Eur Cell Mater 2019; 37: 310–332. 31038192 10.22203/eCM.v037a19

[25] Ryan C Pugliese E Shologu N , et al. The synergistic effect of physicochemical in vitro microenvironment modulators in human bone marrow stem cell cultures. Biomater Adv 2023; 144: 213196. 36455498 10.1016/j.bioadv.2022.213196

[26] Wan H Shin R Chen J , et al. Dextran sulfate-amplified extracellular matrix deposition promotes osteogenic differentiation of mesenchymal stem cells. Acta Biomater 2022; 140: 163–177. 34875356 10.1016/j.actbio.2021.11.049

[27] Rashid R Lim N Chee S , et al. Novel use for polyvinylpyrrolidone as a macromolecular crowder for enhanced extracellular matrix deposition and cell proliferation. Tissue Eng Part C Methods 2014; 20: 994–1002. 24665935 10.1089/ten.tec.2013.0733 PMC4241873

[28] Ang X Lee M Blocki A , et al. Macromolecular crowding amplifies adipogenesis of human bone marrow-derived mesenchymal stem cells by enhancing the pro-adipogenic microenvironment. Tissue Eng Part A 2014; 20: 966–981. 24147829 10.1089/ten.tea.2013.0337 PMC3938936

[29] Lee M Goralczyk A Kriszt R , et al. ECM microenvironment unlocks brown adipogenic potential of adult human bone marrow-derived MSCs. Sci Rep 2016; 6: 21173. 26883894 10.1038/srep21173 PMC4756694

[30] Korntner S Di Nubila A Gaspar D , et al. Macromolecular crowding in animal component-free, xeno-free and foetal bovine serum media for human bone marrow mesenchymal stromal cell expansion and differentiation. Front Bioeng Biotechnol 2023; 11: 1136827. 36949882 10.3389/fbioe.2023.1136827 PMC10025396

[31] Guan S Wu S Li G , et al. Macromolecular crowding facilitates rapid fabrication of intact, robust cell sheets. Biotechnol Lett 2023; 45: 57–67. 36550337 10.1007/s10529-022-03336-w

[32] Zimmermann R Nitschke M Magno V , et al. Discriminant principal component analysis of ToF-SIMS spectra for deciphering compositional differences of MSC-secreted extracellular matrices. Small Methods 2023; 7: e2201157. 10.1002/smtd.202201157 36978251

[33] Prewitz M Stißel A Friedrichs J , et al. Extracellular matrix deposition of bone marrow stroma enhanced by macromolecular crowding. Biomaterials 2015; 73: 60–69. 26398310 10.1016/j.biomaterials.2015.09.014

[34] De Pieri A Korntner S Capella-Monsonis H , et al. Macromolecular crowding transforms regenerative medicine by enabling the accelerated development of functional and truly three-dimensional cell assembled micro tissues. Biomaterials 2022; 287: 121674. 35835003 10.1016/j.biomaterials.2022.121674

[35] Fok SW Gresham RC Ryan W , et al. Macromolecular crowding and decellularization method increase the growth factor binding potential of cell-secreted extracellular matrices. Front Bioeng Biotechnol 2023; 11: 1091157. 36756385 10.3389/fbioe.2023.1091157 PMC9899907

[36] Chiang CE Fang YQ Ho CT , et al. Bioactive decellularized extracellular matrix derived from 3D stem cell spheroids under macromolecular crowding serves as a scaffold for tissue engineering. Adv Healthc Mater 2021; 10: 2100024. 10.1002/adhm.202100024 33890420

[37] Satyam A Kumar P Fan X , et al. Macromolecular crowding meets tissue engineering by self-assembly: a paradigm shift in regenerative medicine. Adv Mater 2014; 26: 3024–3034. 24505025 10.1002/adma.201304428

[38] Gaspar D Fuller K Zeugolis D . Polydispersity and negative charge are key modulators of extracellular matrix deposition under macromolecular crowding conditions. Acta Biomater 2019; 88: 197–210. 30831324 10.1016/j.actbio.2019.02.050

[39] Wang X Ge J Tredget E , et al. The mouse excisional wound splinting model, including applications for stem cell transplantation. Nat Protoc 2013; 8: 302–309. 23329003 10.1038/nprot.2013.002

[40] Fischer K Litmanovich B Sivaraj D , et al. Protocol for the splinted, human-like excisional wound model in mice. Bio Protoc 2023; 13: e4606. 10.21769/BioProtoc.4606 PMC9909311 36816987

[41] Ren L Zhou B Chen L . Silicone ring implantation in an excisional murine wound model. Wounds 2012; 24: 36–42. 25876236

[42] Lam M Nauta A Meyer N , et al. Effective delivery of stem cells using an extracellular matrix patch results in increased cell survival and proliferation and reduced scarring in skin wound healing. Tissue Eng Part A 2013; 19: 738–747. 23072446 10.1089/ten.tea.2012.0480 PMC3566655

[43] Du S Elliman S Zeugolis D , et al. Carrageenan as a macromolecular crowding agent in human umbilical cord derived mesenchymal stromal cell culture. Int J Biol Macromol 2023; 251: 126353. 37591431 10.1016/j.ijbiomac.2023.126353

[44] Guillaumin S Gurdal M Zeugolis D . Gums as macromolecular crowding agents in human skin fibroblast cultures. Life (Basel) 2024; 14: 435. 38672707 10.3390/life14040435 PMC11051389

[45] Rampin A Rossoni A Chaniotaki L , et al. Xenogeneic versus allogeneic serum and macromolecular crowding in human tenocyte cultures. Eur J Cell Biol 2024; 103: 151445. 39024989 10.1016/j.ejcb.2024.151445

[46] Capella-Monsonís H Coentro JQ Graceffa V , et al. An experimental toolbox for characterization of mammalian collagen type I in biological specimens. Nat Protoc 2018; 13: 507–529. 29446773 10.1038/nprot.2017.117

[47] Garnica-Galvez S Korntner S Skoufos I , et al. Hyaluronic acid as macromolecular crowder in equine adipose-derived stem cell cultures. Cells 2021; 10: 859. 33918830 10.3390/cells10040859 PMC8070604

[48] Rampin A Skoufos I Raghunath M , et al. Allogeneic serum and macromolecular crowding maintain native equine tenocyte function in culture. Cells 2022; 11: 1562. 35563866 10.3390/cells11091562 PMC9103545

[49] Capella-Monsonís H De Pieri A Peixoto R , et al. Extracellular matrix-based biomaterials as adipose-derived stem cell delivery vehicles in wound healing: a comparative study between a collagen scaffold and two xenografts. Stem Cell Res Ther 2020; 11: 510. 33246508 10.1186/s13287-020-02021-x PMC7694925

[50] Khorasani H Zheng Z Nguyen C , et al. A quantitative approach to scar analysis. Am J Pathol 2011; 178: 621–628. 21281794 10.1016/j.ajpath.2010.10.019 PMC3070584

[51] Djalali-Cuevas A Rettel M Stein F , et al. Macromolecular crowding in human tenocyte and skin fibroblast cultures: a comparative analysis. Mater Today Bio 2024; 25: 100977. 10.1016/j.mtbio.2024.100977 PMC10846491 38322661

[52] Ryan CN Pugliese E Shologu N , et al. Physicochemical cues are not potent regulators of human dermal fibroblast trans-differentiation. Biomater Biosyst 2023; 11: 100079. 37720487 10.1016/j.bbiosy.2023.100079 PMC10499661

[53] Tsiapalis D Kearns S Kelly JL , et al. Growth factor and macromolecular crowding supplementation in human tenocyte culture. Biomater Biosyst 2021; 1: 100009. 36825160 10.1016/j.bbiosy.2021.100009 PMC9934496

[54] De Pieri A Rana S Korntner S , et al. Seaweed polysaccharides as macromolecular crowding agents. Int J Biol Macromol 2020; 164: 434–446. 32679331 10.1016/j.ijbiomac.2020.07.087

[55] Teleshova N Keller MJ Fernández Romero JA , et al. Results of a phase 1, randomized, placebo-controlled first-in-human trial of griffithsin formulated in a carrageenan vaginal gel. PLoS One 2022; 17: e0261775. 10.1371/journal.pone.0261775 PMC8775213 35051209

[56] Magnan S Tota J El-Zein M , et al. Efficacy of a carrageenan gel against transmission of cervical HPV (CATCH): interim analysis of a randomized, double-blind, placebo-controlled, phase 2B trial. Clin Microbiol Infect 2019; 25: 210–216. 29684633 10.1016/j.cmi.2018.04.012 PMC8274946

[57] Halley C Honeywill C Kang J , et al. Preventing upper respiratory tract infections with prophylactic nasal carrageenan: a feasibility study. Future Microbiol 2023; 18: 1319–1328. 37830932 10.2217/fmb-2021-0122

[58] Diller RB Tabor AJ . The role of the extracellular matrix (ECM) in wound healing: a review. Biomimetics 2022; 7: 87. 35892357 10.3390/biomimetics7030087 PMC9326521

[59] Xue M Jackson CJ . Extracellular matrix reorganization during wound healing and its impact on abnormal scarring. Adv Wound Care (New Rochelle) 2015; 4: 119–136. 25785236 10.1089/wound.2013.0485 PMC4352699

[60] Gardeazabal L Izeta A . Elastin and collagen fibres in cutaneous wound healing. Exp Dermatol 2024; 33: e15052. 10.1111/exd.15052 38483134

[61] Abreu-Velez AM Howard MS . Collagen IV in normal skin and in pathological processes. N Am J Med Sci 2012; 4: 1. 22393540 10.4103/1947-2714.92892 PMC3289483

[62] Ramalingam R Jiang G Larjava H , et al. Macromolecular crowding regulates matrix composition and gene expression in human gingival fibroblast cultures. Sci Rep 2023; 13: 2047. 36739306 10.1038/s41598-023-29252-1 PMC9899282

[63] Vo AN Kundu S Strong C , et al. Enhancement of neuroglial extracellular matrix formation and physiological activity of dopaminergic neural cocultures by macromolecular crowding. Cells 2022; 11: 2131. 35883574 10.3390/cells11142131 PMC9317039

[64] Wan H-Y Chen JCH Xiao Q , et al. Stabilization and improved functionality of three-dimensional perfusable microvascular networks in microfluidic devices under macromolecular crowding. Biomater Res 2023; 27: 32. 37076899 10.1186/s40824-023-00375-w PMC10116810

[65] Garnica-Galvez S Skoufos I Tzora A , et al. Macromolecular crowding in equine bone marrow mesenchymal stromal cell cultures using single and double hyaluronic acid macromolecules. Acta Biomater 2023; 170: 111–123. 37634833 10.1016/j.actbio.2023.08.042

[66] Karayi AK Basavaraj V Narahari SR , et al. Human skin fibrosis: up-regulation of collagen type III gene transcription in the fibrotic skin nodules of lower limb lymphoedema. Trop Med Int Health 2020; 25: 319–327. 31816141 10.1111/tmi.13359

[67] Badid C Vincent M Mcgregor B , et al. Mycophenolate mofetil reduces myofibroblast infiltration and collagen III deposition in rat remnant kidney. Kidney Int 2000; 58: 51–61. 10886549 10.1046/j.1523-1755.2000.00140.x

[68] Giovanini AF Gonzaga CC Zielak JC , et al. Platelet-rich plasma (PRP) impairs the craniofacial bone repair associated with its elevated TGF-β levels and modulates the co-expression between collagen III and α-smooth muscle actin. J Orthop Res 2011; 29: 457–463. 20922797 10.1002/jor.21263

[69] Dominici M Le Blanc K Mueller I , et al. Minimal criteria for defining multipotent mesenchymal stromal cells. The International Society for Cellular Therapy position statement. Cytotherapy 2006; 8: 315–317. 16923606 10.1080/14653240600855905

[70] Assis-Ribas T Forni M Winnischofer S , et al. Extracellular matrix dynamics during mesenchymal stem cells differentiation. Dev Biol 2018; 437: 63–74. 29544769 10.1016/j.ydbio.2018.03.002

[71] Lin C Xin Z Dai J , et al. Commonly used mesenchymal stem cell markers and tracking labels: limitations and challenges. Histol Histopathol 2013; 28: 1109–1116. 23588700 10.14670/hh-28.1109 PMC3839663

[72] Machado Cde V Telles P Nascimento I . Immunological characteristics of mesenchymal stem cells. Rev Bras Hematol Hemoter 2013; 35: 62–67. 23580887 10.5581/1516-8484.20130017 PMC3621638

[73] Choudhery MS Mahmood R Harris DT , et al. Minimum criteria for defining induced mesenchymal stem cells. Cell Biol Int 2022; 46: 986–989. 35293653 10.1002/cbin.11790

[74] Anderson P Carrillo-Gálvez AB García-Pérez A , et al. CD105 (endoglin)-negative murine mesenchymal stromal cells define a new multipotent subpopulation with distinct differentiation and immunomodulatory capacities. PLoS One 2013; 8: e76979. 10.1371/journal.pone.0076979 PMC3790740 24124603

[75] Mark P Kleinsorge M Gaebel R , et al. Human mesenchymal stem cells display reduced expression of CD105 after culture in serum-free medium. Stem Cells Int 2013; 2013: 698076. 24194767 10.1155/2013/698076 PMC3806428

[76] Wang D Liu N Xie Y , et al. Different culture method changing CD105 expression in amniotic fluid MSCs without affecting differentiation ability or immune function. J Cell Mol Med 2020; 24: 4212–4222. 32119193 10.1111/jcmm.15081 PMC7171344

[77] Varma M Breuls R Schouten T , et al. Phenotypical and functional characterization of freshly isolated adipose tissue-derived stem cells. Stem Cells Dev 2007; 16: 91–104. 17348807 10.1089/scd.2006.0026

[78] Yoshimura K Shigeura T Matsumoto D , et al. Characterization of freshly isolated and cultured cells derived from the fatty and fluid portions of liposuction aspirates. J Cell Physiol 2006; 208: 64–76. 16557516 10.1002/jcp.20636

[79] Chen S Liang B Xu J . Unveiling heterogeneity in MSCs: exploring marker-based strategies for defining MSC subpopulations. J Transl Med 2024; 22: 459. 38750573 10.1186/s12967-024-05294-5 PMC11094970

[80] Ma L Huang Z Wu D , et al. CD146 controls the quality of clinical grade mesenchymal stem cells from human dental pulp. Stem Cell Res Ther 2021; 12: 488. 34461987 10.1186/s13287-021-02559-4 PMC8404346

[81] Wu C Liu F Sytwu H , et al. CD146+mesenchymal stem cells display greater therapeutic potential than CD146- cells for treating collagen-induced arthritis in mice. Stem Cell Res Ther 2016; 7: 23. 26841872 10.1186/s13287-016-0285-4 PMC4741021

[82] Harkness L Zaher W Ditzel N , et al. CD146/MCAM defines functionality of human bone marrow stromal stem cell populations. Stem Cell Res Ther 2016; 7: 1–13. 26753846 10.1186/s13287-015-0266-z PMC4710006

[83] Paduano F Marrelli M Palmieri F , et al. CD146 expression influences periapical cyst mesenchymal stem cell properties. Stem Cell Rev Rep 2016; 12: 592–603. 27406247 10.1007/s12015-016-9674-4

[84] Espagnolle N Guilloton F Deschaseaux F , et al. CD146 expression on mesenchymal stem cells is associated with their vascular smooth muscle commitment. J Cell Mol Med 2014; 18: 104–114. 24188055 10.1111/jcmm.12168 PMC3916122

[85] Al Bahrawy M . Comparison of the migration potential through microperforated membranes of CD146+ GMSC population versus heterogeneous GMSC population. Stem Cells Int 2021; 2021: 5583421. 33777147 10.1155/2021/5583421 PMC7979285

[86] Zhang L Sun Y Zhang X , et al. Comparison of CD146+/-mesenchymal stem cells in improving premature ovarian failure. Stem Cell Res Ther 2022; 13: 267. 35729643 10.1186/s13287-022-02916-x PMC9209844

[87] Kuçi Z Piede N Vogelsang K , et al. Expression of HLA-DR by mesenchymal stromal cells in the platelet lysate era: an obsolete release criterion for MSCs? J Transl Med 2024; 22: 39. 38195462 10.1186/s12967-023-04684-5 PMC10775607

[88] Romieu-Mourez R François M Boivin M-N , et al. Regulation of MHC class II expression and antigen processing in murine and human mesenchymal stromal cells by IFN-γ, TGF-β, and cell density. J Immunol 2007; 179: 1549–1558. 17641021 10.4049/jimmunol.179.3.1549

[89] Bocelli-Tyndall C Trella E Frachet A , et al. FGF2 induces RANKL gene expression as well as IL1β regulated MHC class II in human bone marrow-derived mesenchymal progenitor stromal cells. Ann Rheum Dis 2015; 74: 260–266. 24249810 10.1136/annrheumdis-2013-204235

[90] Bocelli-Tyndall C Zajac P Di Maggio N , et al. Fibroblast growth factor 2 and platelet-derived growth factor, but not platelet lysate, induce proliferation-dependent, functional class II major histocompatibility complex antigen in human mesenchymal stem cells. Arthritis Rheum 2010; 62: 3815–3825. 20824797 10.1002/art.27736

[91] Grau-Vorster M Laitinen A Nystedt J , et al. HLA-DR expression in clinical-grade bone marrow-derived multipotent mesenchymal stromal cells: a two-site study. Stem Cell Res Ther 2019; 10: 164. 31196185 10.1186/s13287-019-1279-9 PMC6567533

[92] Zeiger A Loe F Li R , et al. Macromolecular crowding directs extracellular matrix organization and mesenchymal stem cell behavior. PLoS One 2012; 7: e37904. 10.1371/journal.pone.0037904 PMC3359376 22649562

[93] Janmey P Hinz B McCulloch C . Physics and physiology of cell spreading in two and three dimensions. Physiology (Bethesda) 2021; 36: 382–391. 34704856 10.1152/physiol.00020.2021 PMC8560373

[94] Yang Y Wang X Wang Y , et al. Influence of cell spreading area on the osteogenic commitment and phenotype maintenance of mesenchymal stem cells. Sci Rep 2019; 9: 6891. 31053728 10.1038/s41598-019-43362-9 PMC6499796

[95] Eliopoulos N Stagg J Lejeune L , et al. Allogeneic marrow stromal cells are immune rejected by MHC class I- and class II-mismatched recipient mice. Blood 2005; 106: 4057–4065. 16118325 10.1182/blood-2005-03-1004

[96] Sheldon S Hasleton P Yonan N , et al. Rejection in heart transplantation strongly correlates with HLA-DR antigen mismatch. Transplantation 1994; 58: 719–722. 7940693

[97] Ozdemir B Aksoy P Haberal A , et al. Relationship of HLA-DR expression to rejection and mononuclear cell infiltration in renal allograft biopsies. Ren Fail 2004; 26: 247–251. 15354973 10.1081/jdi-200026752

[98] Ding D Chou H Chang Y , et al. Characterization of HLA-G and related immunosuppressive effects in human umbilical cord stroma-derived stem cells. Cell Transplant 2016; 25: 217–228. 26044082 10.3727/096368915X688182

[99] Technau A Froelich K Hagen R , et al. Adipose tissue-derived stem cells show both immunogenic and immunosuppressive properties after chondrogenic differentiation. Cytotherapy 2011; 13: 310–317. 20795757 10.3109/14653249.2010.504769

[100] Du W Reppel L Leger L , et al. Mesenchymal stem cells derived from human bone marrow and adipose tissue maintain their immunosuppressive properties after chondrogenic differentiation: role of HLA-G. Stem Cells Dev 2016; 25: 1454–1469. 27465875 10.1089/scd.2016.0022

[101] Zuber P Kuppner M De Tribolet N . Transforming growth factor-beta 2 down-regulates HLA-DR antigen expression on human malignant glioma cells. Eur J Immunol 1988; 18: 1623–1626. 3142781 10.1002/eji.1830181023

[102] Lukomskyj AO Rao N Yan L , et al. Stem cell-based tissue engineering for the treatment of burn wounds: a systematic review of preclinical studies. Stem Cell Rev Rep 2022; 18: 1926–1955. 35150392 10.1007/s12015-022-10341-z PMC9391245

[103] Maxson S Lopez E Yoo D , et al. Concise review: role of mesenchymal stem cells in wound repair. Stem Cells Transl Med 2012; 1: 142–149. 23197761 10.5966/sctm.2011-0018 PMC3659685

[104] Isakson M de Blacam C Whelan D , et al. Mesenchymal stem cells and cutaneous wound healing: current evidence and future potential. Stem Cells Int 2015; 2015: 831095. 26106431 10.1155/2015/831095 PMC4461792

[105] Huang Y Gou M Da L , et al. Mesenchymal stem cells for chronic wound healing: current status of preclinical and clinical studies. Tissue Eng Part B Rev 2020; 26: 555–570. 32242479 10.1089/ten.TEB.2019.0351

[106] Liu H Yang R Zhao S , et al. Collagen scaffolds derived from bovine skin loaded with MSC optimized M1 macrophages remodeling and chronic diabetic wounds healing. Bioeng Transl Med 2023; 8: e10467. 10.1002/btm2.10467 PMC10189465 37206210

[107] Geesala R Bar N Dhoke NR , et al. Porous polymer scaffold for on-site delivery of stem cells–protects from oxidative stress and potentiates wound tissue repair. Biomaterials 2016; 77: 1–13. 26576045 10.1016/j.biomaterials.2015.11.003

[108] Falanga V Iwamoto S Chartier M , et al. Autologous bone marrow-derived cultured mesenchymal stem cells delivered in a fibrin spray accelerate healing in murine and human cutaneous wounds. Tissue Eng 2007; 13: 1299–1312. 17518741 10.1089/ten.2006.0278

[109] Cerqueira M Pirraco R Santos T , et al. Human adipose stem cells cell sheet constructs impact epidermal morphogenesis in full-thickness excisional wounds. Biomacromolecules 2013; 14: 3997–4008. 24093541 10.1021/bm4011062

[110] Ochiai D Abe Y Fukutake M , et al. Cell sheets using human amniotic fluid stem cells reduce tissue fibrosis in murine full-thickness skin wounds. Tissue Cell 2021; 68: 101472. 33360545 10.1016/j.tice.2020.101472

[111] Di Nubila A Doulgkeroglou M Gurdal M , et al. In vitro and in vivo assessment of a non-animal sourced chitosan scaffold loaded with xeno-free umbilical cord mesenchymal stromal cells cultured under macromolecular crowding conditions. Biomater Biosyst 2024; 16: 100102. 40225717 10.1016/j.bbiosy.2024.100102 PMC11993840

[112] Di Nubila A Doulgkeroglou M Gurdal M , et al. Corrigendum to “In vitro and in vivo assessment of a non-animal sourced chitosan scaffold loaded with xeno-free umbilical cord mesenchymal stromal cells cultured under macromolecular crowding conditions” [Biomaterials and biosystems, volume 16, December 2024, 100102]. Biomater Biosyst 2025; 17: 100106. 40201907 10.1016/j.bbiosy.2025.100106 PMC11976480

[113] Davidson J Yu F Opalenik S . Splinting strategies to overcome confounding wound contraction in experimental animal models. Adv Wound Care (New Rochelle) 2013; 2: 142–148. 2014/02/15.. 24527337 10.1089/wound.2012.0424 PMC3656626

[114] Tang JN Halaseh FF Khan N , et al. 3D-printed wound chambers: a novel splint system wound healing research. Wounds Int 2021; 12: 54–58.

[115] Baek S Jang U Shin J , et al. Shape memory alloy as an internal splint in a rat model of excisional wound healing. Biomed Mater 2021; 16: 025002. 33429379 10.1088/1748-605X/abda89

[116] XiaoMing X Yan C JiaMing G , et al. Human umbilical cord mesenchymal stem cells combined with porcine small intestinal submucosa promote the healing of full-thickness skin injury in SD rats. Future Sci OA 2024; 10: FSO955. 10.2144/fsoa-2023-0123 PMC11137796 38817375

[117] Jorgensen A Gorkun A Mahajan N , et al. Multicellular bioprinted skin facilitates human-like skin architecture in vivo. Sci Transl Med 2023; 15: eadf7547. 10.1126/scitranslmed.adf7547 37792956

